# Supplementary material for: The causal role of circulating inflammatory markers in osteoporosis: a bidirectional Mendelian randomized study
Source: Front Immunol. 2024 Jul 18;15:1412298. doi: 10.3389/fimmu.2024.1412298 (PMC11291241; doi:10.3389/fimmu.2024.1412298)
Supplement: Supplementary file 1 [file DataSheet_1.zip › supplementary material/OSTEOPOROSIS-CIM/GCST90274839.tsv.gz_forest.pdf]

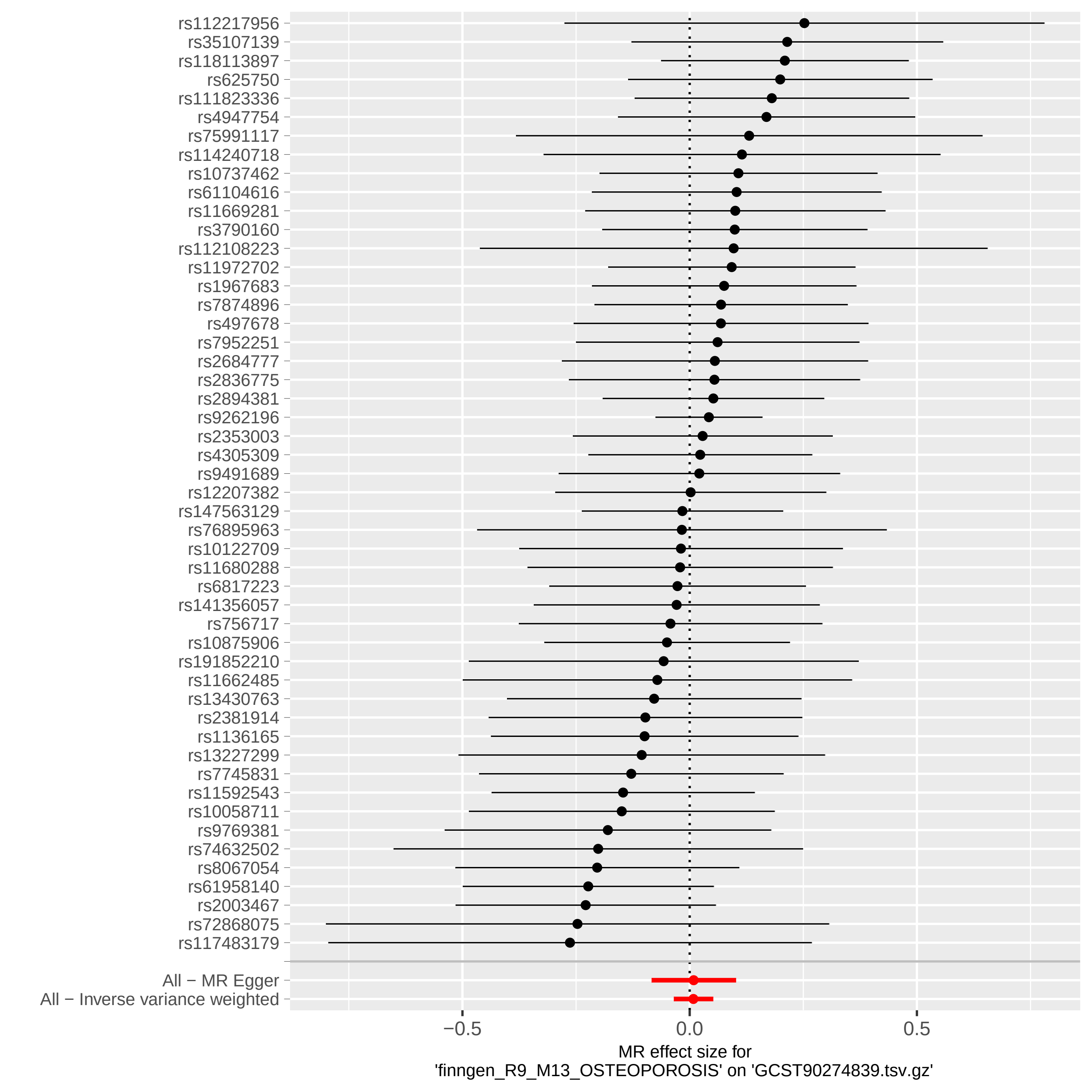
rs112217956
rs35107139
rs118113897
rs625750
rs111823336
rs4947754
rs75991117
rs114240718
rs10737462
rs61104616
rs11669281
rs3790160
rs112108223
rs11972702
rs1967683
rs7874896
rs497678
rs7952251
rs2684777
rs2836775
rs2894381
rs9262196
rs2353003
rs4305309
rs9491689
rs12207382
rs147563129
rs76895963
rs10122709
rs11680288
rs6817223
rs141356057
rs756717
rs10875906
rs191852210
rs11662485
rs13430763
rs2381914
rs1136165
rs13227299
rs7745831
rs11592543
rs10058711
rs9769381
rs74632502
rs8067054
rs61958140
rs2003467
rs72868075
rs117483179
All − MR Egger
All − Inverse variance weighted
−0.5
0.0
0.5
MR effect size for
'finngen_R9_M13_OSTEOPOROSIS' on 'GCST90274839.tsv.gz'
